# Asthma incidence and risk factors in a national longitudinal sample of adolescent Canadians: a prospective cohort study

**DOI:** 10.1186/1471-2466-14-51

**Published:** 2014-03-25

**Authors:** Joshua A Lawson, Ian Janssen, Mark W Bruner, Alomgir Hossain, William Pickett

**Affiliations:** 1 Canadian Centre for Health and Safety in Agriculture, Royal University Hospital, University of Saskatchewan, Saskatoon, SK, Canada; 2 Department of Medicine, University of Saskatchewan, Saskatoon, SK, Canada; 3 School of Kinesiology and Health Studies, Queen’s University, Kingston, ON, Canada; 4 Department of Public Health Sciences, Queen’s University, Kingston, ON, Canada; 5 School of Physical and Health Education, Nipissing University, North Bay, ON, Canada; 6 Department of Community Health and Epidemiology, Saskatchewan Health Quality Council, University of Saskatchewan, Saskatoon, SK, Canada; 7 Department of Emergency Medicine, Queen’s University, Kingston, ON, Canada

**Keywords:** Asthma, Adolescents, Sex, Incidence, Urban–rural, Cohort effect, Environmental tobacco smoke

## Abstract

**Background:**

Estimates of asthma incidence and its possible determinants in adolescent populations have rarely been obtained using prospective designs. We sought to identify socio-demographic and other patterns in the incidence of asthma among Canadian adolescents and to examine possible behavioural and environmental determinants of asthma incidence using longitudinal analyses.

**Methods:**

We used data from the National Population Health Survey (NPHS), a nationally representative longitudinal survey of Canadians. All persons aged 12–18 years without asthma at baseline were followed up to a maximum of 12 years. The outcome was a reported diagnosis of asthma during the follow-up period. Analyses were weighted to the population and bootstrapping procedures were used to estimate variances.

**Results:**

Participants (n = 956) represented 2,038,890 adolescents of whom 293,450 (14.4%) developed asthma over the 21,274,890 person-years of follow-up. Overall, the incidence of asthma was 10.2 per 1000 person-years. In adjusted Cox regression analysis, being female (HR = 2.13, 95% CI = 1.26-3.62, p = 0.005) and being exposed to passive smoking (HR = 2.06, 95% CI = 1.27-3.34, p = 0.003) were associated with the development of asthma while no statistically significant associations were identified for rural residence, being overweight, and other health behaviours. There was also an apparent cohort effect among girls where girls who were older at baseline reported being diagnosed with asthma more over the follow-up than their younger counterparts. This was not observed among males.

**Conclusions:**

Asthma prevention initiatives for adolescents should target girls and focus on smoking exposures. The role that differential diagnostic patterns play in these observations should be investigated to more accurately assess the incidence of asthma.

## Background

Asthma imposes a large burden on the individual and on health care systems. [1] Currently, asthma prevalence is approximately 10% globally [2] and 13% within Canada [3] with some Canadian centers reaching a 19% childhood asthma prevalence [4]. Unexplained temporal [5-7] and geographic variations [2,8] in asthma prevalence have also been reported with asthma prevalence increasing over the past few decades and higher asthma prevalence in Westernized nations. Asthma prevalence is substantially higher in children than in adults [9] and varies by sex where in children it is higher among boys, although this reverses around puberty [5,9]. While asthma and wheeze phenotypes [10] and the natural history of disease with identification of environmental risk factors [11-15] has been frequently studied in younger children, adolescents remain a largely understudied population with regard to asthma and its potential determinants. Given the difference in disease timing, there are likely different underlying etiologies in the adolescent developmental period compared to earlier development.

In order to better understand the patterns of asthma frequency and etiology of the disease, longitudinal studies with a focus on asthma incidence as opposed to prevalence are preferred. There is a dearth of prospective studies investigating asthma, especially in adolescents. There has been variable reports of asthma incidence among adolescents ranging from 5.2 per 1000 person-years [16] to 24.6 per 1000 person-years [17]. Several potential explanations are of interest including differences in diagnostic practices, engagement in health risk behaviours, and aspects of the environment. More specifically, better understanding the sex differences in asthma during adolescence in light of the apparent “gender switch” that occurs at this time through the use of longitudinal designs and investigation of environmental effects on the development of asthma would help explain recent trends and lead to programs that may prevent asthma development.

As outlined, there are gaps in the literature regarding the etiology of asthma in adolescent populations resulting from a lack of longitudinal research. To help address these gaps, our objectives were to: (1) describe the incidence of asthma within a national sample of Canadian adolescents and (2) identify determinants of asthma incidence among Canadian adolescents with a focus on sex, location of residence, health behaviours, and overweight.

## Methods

### Data source, study design and study population

Data for this study were obtained from the National Population Health Survey (NPHS), [18] a longitudinal health research survey conducted nationally by Statistics Canada. Sampling is designed to be representative of households across the country. Exclusions were people living on Crown lands, residents of Indian reserves, residents of health institutions, full time members of the Canadian Forces Bases, and some remote areas in Ontario and Quebec. Subjects were selected through a stratified, two-stage sampling design (clusters, then dwellings). At the baseline survey in 1994/1995 (Cycle 1), an interviewer administered the questionnaire to one household member, 12 years or older who was chosen to be the longitudinal respondent. This person was selected at random from those persons 12 years and older living in the household. The respondent provided all personal information at each cycle although proxy reporting was allowed for reasons of illness or incapacity. This household member was re-contacted every two years up to 12 years of follow-up (Cycle 7).

At baseline, 17,276 individuals were included as part of the longitudinal panel (participation rate = 83.6%) [18]. The follow-up rate was 92.8% at two years of follow-up and 77.0% at 12 years of follow-up [18]. For the current analysis, participants were 12–18 years of age at baseline. Only those participants without a previous diagnosis of asthma and who had complete information on the variables of interest were included. Ethical approval for data collection was completed by Statistics Canada (Government of Canada). When Statistics Canada sets up a survey, the impact of the survey on responders is taken into account. Researchers working on the project sign an oath of office by which they swear they will faithfully and honestly fulfil their duties as an employee of Statistics Canada in conformity with the requirements of the Statistics act to preserve data confidentiality. Our access to this data was only through approved Research Data Centers following a rigorous screening process and approval of the proposed research in order to use this de-identified data.

### Operational definitions

#### Asthma

The presence of asthma was based on “Has [NAME] ever had asthma that has been diagnosed by a health professional?” If there was a positive response to this question at baseline the person was excluded. All subsequent positive responses to this question during follow-up indicated an incident asthma case. As there is no gold standard in the diagnosis of asthma, questionnaire report of a doctor’s diagnosis of asthma is very frequently used in epidemiological studies. This method has reasonable levels of agreement with other methods of assessment and has been considered the method of choice for large epidemiologic studies [19].

#### Socio-demographic variables

Age, sex, and income status were considered. Age was classified into three groups based on baseline age (12–13 years, 14–15 years, and 16–18 years). Participants were also classified by cohort, determined by year of birth (1981–1982; 1979–1980; or 1976–1978). Income at baseline was assessed based on a variable derived by Statistics Canada using the total income before taxes and deductions of all household members from all sources in the past 12 months as well as the number of persons living in the household. It was categorized as high, middle, and low.

#### Location of residence

Geographic status of residence at baseline was classified as urban vs. rural. Areas with a population of less than 1000 people or with a population density of <400 people/km^2^ were considered rural. This is a commonly used indicator for rural based on Canadian Census Rural Areas which accounts for population size and density [20]. It has been used in previous asthma studies [21].

#### Overweight

Height and weight were self-reported at baseline. From this, baseline BMI was calculated (kg/m^2^) and classified into normal or overweight (including obese) using criteria recommended by the International Obesity Task Force [22] which classify overweight in adolescents using age-sex-specific BMI cut-points that correspond to a BMI of 25 at entry into adulthood.

#### Smoking exposures

Exposure to passive smoking was assessed based on the question “Does anyone in this household smoke regularly inside the house?” A positive response indicated exposure to passive smoking. Personal smoking status was based on the questions “At the present time, do you smoke cigarettes daily, occasionally, or not at all?” In addition to this, a question was asked regarding past smoking. Personal smoking was then classified as never/former and occasionally/daily in order to describe current smoking status (i.e. non-smoker vs. current smoker).

#### Physical activity

Baseline physical activity was based on the reported frequency and duration of different activities that the participant engaged in. Based on this information and the intensity of these activities, energy expenditure was calculated and participants were classified into active and inactive groups. Categorization was based on a cutoff of 6 kcal/kg/day which is considered “active” from guidelines for healthy growth and development of children [23,24].

### Statistical analysis

Statistical analyses were completed using Stata (StataCorp LP, College Station, TX, USA). Throughout all analyses, population weights were applied. Bootstrapping methods were used to estimate the variability around the estimates. Overall and category specific incidence rates of asthma were determined. These patterns were further explored by sex and cohort of birth among those aged 12–17 years to maintain consistency of ages between cycles of follow-up. In this analysis, asthma incidence for each sex-cohort combination was calculated using the outcome at each cycle of follow-up.

Survival analyses were completed to examine associations between the independent variables and time to asthma diagnosis. Cumulative hazard curves were estimated and plotted for each independent variable. Crude and adjusted Cox regression analyses were conducted. Adjusted analyses included each independent variable of interest (sex, age group, location of residence, overweight status, personal smoking, passive smoking exposure, physical activity, and income). Associations were expressed as hazard ratios (HR) and 95% confidence intervals (CI). As part of our diagnostic testing, we tested the proportional hazards assumption for the Cox regression model used in our analysis. This assumption was met with the exception of personal smoking, which showed some evidence of a possible violation. However, important differences in interpretation were not observed.

## Results

There were a total of 1,138 respondents aged 12–18 years at baseline. Of these, 132 had asthma at baseline and were excluded. After excluding those with missing data or who had no follow-up, 956 participants were included. This group represented 2,038,890 young people. All remaining analyses were based on the weighted sample. Table 1 shows the distribution of the variables of interest and comparisons between those who were included and excluded from the analyses. Those included were more likely to be in the oldest age group and less likely to be overweight.

**Table 1:** Comparison of subjects who were included to those who were excluded due to missing data

	**Included**	**Excluded**	p-value*
	**(n = 2,038,890)**	**(n = 443,720)**	
	**%**	**%**	
Sex			
Male	50.0	53.3	
Female	50.0	46.7	0.51
Age group			
12-13	27.3	31.9	
14-15	28.3	36.3	
16-18	44.4	31.8^†^	0.03
Area of residence			
Urban	80.9	81.0	
Rural	19.1	19.0	0.97
BMI			
Normal	80.0	64.5	
Overweight or obese	20.0	35.5*	<0.001
Passive smoking exposure			
Absent	59.5	63.5	
Present	40.5	36.6	0.44
Personal smoking			
Never/Former	83.1	87.1	
Occasionally/Daily	16.9	12.9	0.29
Physical exercise			
Inactive	85.6		
Active	14.4^†^		
Income			
High	15.7	16.1	
Middle	66.8	58.4	
Low	17.4	25.5	0.27

*Comparing those who were included to those who were excluded.

^†^No information was presented for the excluded population due to low cell counts.

The overall incidence of asthma from the 21,274,890 person-years of follow-up was 10.2 cases per 1000 person-years. Table 2 presents the incidence rates by the independent variables of interest. Females had a higher asthma incidence than males (13.2 vs. 6.6 per 1000 person-years). Those with passive smoking exposure were more likely to report a new diagnosis of asthma than those without (13.8 vs. 7.2 per 1000 person-years). While adolescents from rural areas were less likely to report a diagnosis of asthma than urban adolescents over the study period (6.4 vs. 10.7 per 1000 person-years), the differences were not statistically significant (p = 0.22). There were not significant differences in asthma incidence according to overweight (p = 0.40), personal smoking (p = 0.89), and physically active (p = 0.74) status.

**Table 2:** Asthma incidence and results from Cox regression analysis by baseline socio-demographic and health behavior characteristics

	**Person-years of follow-up**	**Number of asthma cases**	**Incidence per 1000 person-years (95% CI)**	**Crude HR (95% CI)**	Adjusted HR* (95% CI)
Sex					
Male	10,766,510	70,770	6.6 (4.5-9.8)	1.00	1.00
Female	10,508,380	138,290	13.2 (9.7-18.2)	1.99 (1.23-3.23)^†^	2.13 (1.26-3.62)
Age group					
12-13	6,007,840	53,130	8.8 (5.6-14.7)	1.00	1.00
14-15	6,044,580	56,290	9.3 (6.0-15.1)	1.03 (0.53-2.00)	1.09 (0.55-2.16)
16-18	9,222,480	99,640	10.8 (7.6-15.8)	1.18 (0.65-2.16)	1.16 (0.63-2.16)
Area of residence					
Urban	17,056,720	182,260	10.7 (8.3-14.0)	1.00	1.00
Rural	4,218,170	26,810	6.4 (3.4-13.2)	0.61 (0.27-1.35)	0.58 (0.25-1.35)
Income					
High	3,363,690	37,290	11.1 (6.4-20.9)	1.00	1.00
Middle	14,358,250	123,840	8.6 (6.5-11.7)	0.78 (0.40-1.54)	0.72 (0.36-1.44)
Low	3,552,950	47,940	13.5 (7.6-26.2)	1.19 (0.53-2.69)	1.13 (0.48-2.66)
BMI status					
Normal	16,839,660	174,930	10.4 (8.0-13.7)	1.00	1.00
Overweight or obese	4,435,230	34,130	7.7 (4.6-14.3)	0.76 (0.39-1.45)	0.73 (0.37-1.46)
Passive smoking exposure					
Absent	12,860,340	93,130	7.2 (5.0-11.0)	1.00	1.00
Present	8,414,550	115,930	13.8 (10.2-18.9)	1.88 (1.19-2.98)^†^	2.06 (1.27-3.34)
Personal smoking					
Never/Former	17,730,630	75,530	9.9 (7.6-13.1)	1.00	1.00
Occasionally/Daily	3,544,260	33,530	9.5 (5.4-17.7)	0.95 (0.48-1.87)	0.67 (0.34-1.30)
Physical exercise					
Inactive	18,263,280	175,820	9.6 (7.4-12.7)	1.00	1.00
Active	3,011,620	33,250	11.0 (6.2-21.1)	1.13 (0.55-2.35)	1.27 (0.57-2.83)

*Adjusted for each variable in the table.

†Signifies p<0.05.

**Figure 1:**
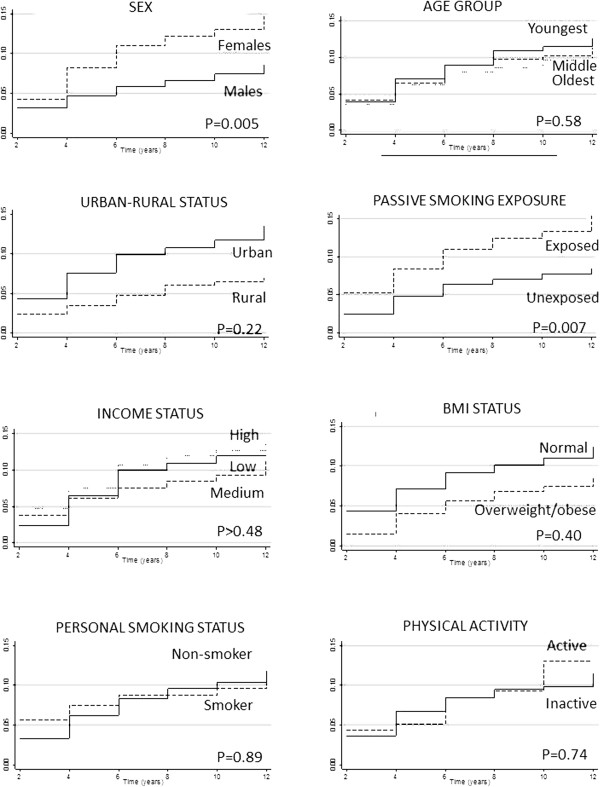
**Cumulative hazard curves for each independent variable of interest.** Solid line – The reference group (with the exception of urban–rural). Dashed line – Comparison group 1. Dotted line – Comparison group 2 (where appropriate).

Figure 1 displays the cumulative hazard curves for asthma incidence by the independent variables of interest. Both crude and adjusted results from Cox regression analyses (Table 2) confirm the descriptive results. After adjustment for potential confounders, there was over a twofold increase in having a diagnosis of asthma among females compared to males (HR = 2.13, 95% CI = 1.26-3.62), and a similar increase among those with passive smoking exposure compared to those without passive smoking exposure (HR = 2.06, 95% CI = 1.27-3.34). There was suggestion that rural residence was protective for asthma compared to urban residence, however, this effect estimate did not reach statistical significance (HR = 0.58, 95% CI = 0.25-1.35).

**Figure 2:**
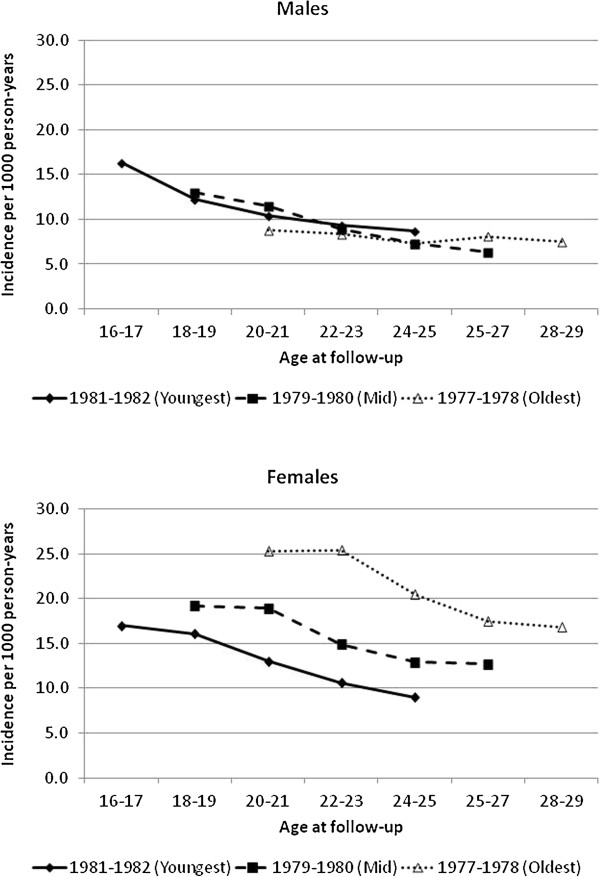
**Asthma incidence at age of follow-up by sex and year of birth for three cohorts.** Diamond with solid line – 1981–1982 (Youngest). Square with dashed line – 1979–1980 (Mid). Triangle with dotted line – 1977–1978 (Oldest).

We plotted the incidence of asthma based on the outcome at each cycle of follow-up and age at that cycle after stratifying by sex and birth cohort (Figure 2). For both males and females, asthma incidence decreased as age at follow-up increased. Among males, the incidence rates at a specific age of follow-up were fairly similar regardless of birth cohort. However, among females, there appeared to be a cohort effect with the oldest cohort having the highest incidence of asthma at each age of follow-up, followed by the middle aged cohort then the youngest cohort. Despite these differences, when we statistically tested the interaction between cohort group and sex, the results were not statistically significant (p > 0.05). The incidence rates for a given age were similar between the youngest female cohort and their male counterparts.

## Discussion

In this representative sample of Canadian adolescents, the incidence of asthma was higher among girls than boys. However, a cohort effect was observed among girls whereby those who were older at baseline had a higher incidence of asthma for a given age at follow-up compared to younger girls. Also, adolescents with passive smoking exposure were at increased risk for diagnosis of asthma compared to those without passive smoking exposure. Finally, there was not a statistically significant association between the incidence of asthma and location of residence, overweight status or physical activity level, although some protection from a rural environment was possible.

The incidence of asthma among adolescents has been studied infrequently. Our incidence rates were higher than those seen in another Canadian study where the incidence among 10–14 year olds followed for 2 years was between 5.2 and 5.8 per 1000 person-years [16]. In a US study, asthma incidence in Grades 4, 7, and 10 students who were followed until high school graduation was 24.6 per 1000 person-years [17]. Differences between incidence rates reported in these studies may reflect differences in study design, sampling, asthma definitions, and length of follow-up. The Canadian study made use of administrative databases and used an asthma case definition based on at least 2 asthma physician visits within 2 years and/or at least 1 hospitalization [16]. While it looked at children and adults, the closest comparable age group with results that we could compare to was 10–14 years which was generally younger than our group. Our youngest age group had a slightly higher asthma incidence rate (8.8 per 1000 person-years). In the US study, children were recruited from the 4th, 7th, and 10^th^ grades with the majority of participants at baseline aged 7–9 years, then followed until high school graduation, which may have limited the length of follow-up in the older groups [17]. Also, the study was designed to investigate air pollution and included a relatively targeted population in order to maximize the variability of pollutant profiles, include demographic stability, likely parental cooperation, and appropriate linkages between elementary, middle, and secondary schools [25].

A recent study from Spain investigating asthma incidence in two cohorts (adolescents aged 11–16 years and adults ages 20–44 years) reported a higher incidence over 9 years in children compared to adults (15.69 cases per 1000 person-years vs. 4.76 cases per 1000 person-years, respectively) [26]. While our study did not consider adults at baseline, we found that the older adolescents had a higher overall incidence than the younger adolescents at baseline, somewhat contradicting the results of Pereira et al. In addition to this, our incidence rates overall were slightly lower than those reported previously. These differences were likely due to the higher incidence in the earlier cohort of females given the observed cohort effect. Despite these differences, when considering incidence at a specific age of follow-up, in support of the results of Pereira et al., we found that as age increases, asthma incidence decreased.

As expected based on studies of asthma prevalence, which show a sex reversal around puberty, we found that adolescent girls were more at risk for developing asthma than adolescent boys. Several reasons have been suggested for this sex reversal including hormonal changes around puberty [27]. We also identified a potential cohort effect among females that was not apparent in males. Given the low likelihood that changes in biological response or environmental exposure could occur so quickly, reporting and diagnostic labeling patterns are the most likely explanations.

In a study of 12–15 year olds from Denmark, girls were at increased risk of underdiagnosed asthma compared to boys and most of these females did not present to a physician [28]. This study was completed in the early 1990’s. Since this time, it has been shown that among adults, recent changes in asthma epidemiology may result from increased symptom awareness and willingness to report symptoms as well as an increased willingness of physicians to diagnosis asthma [29]. While there was an increase in the prevalence of asthma symptoms and diagnosis between 1992/93 and 1998/9 there was a reduced risk of hyper-responsiveness on methacholine challenge over this time period. Over these six years, which is the difference in time between our three age cohorts and less time than the follow-up period of our study, there was a significant increase in the perception of bronchoconstriction [29]. It could also be that the oldest cohort of females entering the cohort had asthma at the outset that was undiagnosed. This could result in a “catch-up” period with more new diagnoses in the older cohorts as females moved through adolescence into adulthood while girls in the youngest cohort would already have been diagnosed pre-adolescence.

Another explanation is that recently there has been an increase in the recognition that different asthma phenotypes may play a role in the recent asthma patterns. Differences in presentation affect the diagnosis of asthma and this has been seen to result in differences in diagnostic patterns between males and females with females presenting with patterns that are less recognizable as typical asthma patterns in youth such as nocturnal cough without wheeze while there were no differences in diagnostic patterns when the child was atopic, a more typical asthma characteristic among children [30]. This is in line with evidence in young adults showing that most cases of new asthma among women are non-allergic cases [31]. What is more compelling, is that recent evidence from adults living in Italy suggest that while asthma prevalence was stable through the 1990’s, it again increased in the 2000’s with most of this among those without allergic rhinitis (i.e. non-allergic) [32]. This, combined with further evidence from Italy, which shows that there has been increasing prevalence of allergic related conditions such as atopic eczema in children and adolescents with a slight increase in ever asthma prevalence in adolescents, [33] suggest that younger females may be more likely to be diagnosed earlier due to allergic presentation, explaining the similar asthma incidence as males in the youngest cohort. Due to the more recent understanding that different phenotypes should be recognized as asthma, a non-allergic phenotype may account for the increased diagnosis in the older cohorts of our study, whom should have been diagnosed earlier.

The aforementioned trends may not occur in boys due to earlier diagnosis of asthma as seen by a higher asthma prevalence among boys in childhood. However, some caution should be used in the interpretation of this result given the lack of statistical significance in the testing of interaction between the cohort group and sex. Despite this, the fact that the observed trends occur consistently across age cohorts with a clear decrease in incidence in a dose–response fashion from the oldest to the youngest cohort among females and that there are very stable patterns among males suggest that this is likely a true epidemiologic pattern as opposed to an artifact resulting from the design or methodology.

We did not identify a statistically significant association between location of residence and the incidence of asthma, although the protective effects of rural dwellings were in the expected direction. It could be that the definition of rural we used was not specific enough, and that farming exposures, which were not measured in the NPHS, are more important than the general rural environment. Alternatively, the protective effects of rural or farming environments may result from early life exposures [34] and our assessment did not capture the appropriate window of exposure or that the early protective effect of rural living becomes weaker in adolescence. In one longitudinal study of Canadian children aged 0–11 years at baseline, living in a farming environment was associated with less asthma but living in a rural-non-farming environment was not [35]. Finally, given that we observed the direction of the effect we expected to see, there could be uncontrolled confounding that remains poorly understood or other factors that we did not consider that may mediate the mechanism involved.

Passive tobacco smoke exposure was associated with asthma incidence. This association is well-established and we confirm it in an adolescent population using a longitudinal design. We did not, however, identify a similar association between personal smoking and asthma. It may be that adolescents who were predisposed to lung disease or who have had minor symptoms in the past but without a diagnosis of asthma avoided personal smoking.

Results from recent cross-sectional studies of children have provided consistent evidence that there is an association between asthma and obesity [36-38]. However, it has been difficult to interpret the relationship in a causal sense given the lack of temporality inherent in cross-sectional studies. Assessment of the results from longitudinal studies has had its own challenges due to heterogeneity in study completion including differences in outcome and exposure definitions, adjustment of potential confounders, and characteristics of study populations. We did not observe an association between health behaviours or overweight with asthma.

There are several limitations that should be considered. While the study was designed to be representative of the national population of adolescents and population weights were applied to all analyses, the analyses were based on a relatively small un-weighted sample. A small un-weighted sample size may not truthfully represent such a large sample. However, this weighted analysis was necessary given the sample selection procedures and administrative protocol. Several efforts were also made to ascertain data accuracy and account for it in the sampling weights [18].

The primary endpoint of interest was the report of incident doctors diagnosed asthma. Despite the common and accepted use of questionnaire report in this ascertainment of outcome, it is possible that there is some misclassification. This seems unlikely due to the use of the questionnaire given that sensitivity and specificity are high when comparing questionnaire report to blinded physician assessment [39], respiratory symptoms have been found to be accurately reported by the adolescent and strongly agree with parental reports in terms of asthma diagnoses. [40], and there was a short recall period throughout the follow-up (maximum of 2 years). However, while the misclassification of asthma because of the mode of data collection (interviewed questionnaire report) is unlikely there may still be misclassification of true asthma status. It may be that the participant did not seek medical attention and was therefore unable to receive an asthma diagnosis. It may also be that the physician labeled the participant with asthma when they did not have it as observed by Aaron et al. [41] who found that there may be substantial overdiagnosis of asthma in Canadian adults. Both of these situations may result in misclassification.

The NPHS was designed to investigate a broad spectrum of health outcomes, limiting the information collected specific to lung health and important to consider including atopy, puberty onset, or mold exposure. Despite this, we were able to examine socio-demographics, key environmental exposures (i.e. smoking and passive smoking), and location of dwelling.

This study also has many strengths and practical implications. NPHS was designed in order to ensure a sample that is representative of Canadian adolescents. This makes the study generalizable to the Canadian public and likely to other populations internationally. The NPHS participation rates were excellent at baseline and over the 12 years of follow-up, reducing the likelihood of biased associations due to selection processes and suggesting that statistical power is a more likely methodological explanation of a lack of expected findings. This also speaks to the prolonged investment of time and commitment by the Statistics Canada study personnel, study participants, and study community with an interest in public health. We have conducted one of only a few analyses that investigated patterns of incidence and risk factors for asthma among adolescents. Based on our results, we suggest that programs should continue to be implemented promoting the banning of cigarette smoking in public places. Research into the sex differences, whether they are biological or behavioural (i.e., diagnostic patterns, patterns of disease presentation, etc.) is of utmost importance to ensure adequate management of asthma in all groups. There should be continued focus on the study of the associations between asthma and obesity and location of residence as well as other health behaviours.

## Conclusions

In conclusion, among a population of Canadian adolescents we found that the incidence of asthma differed by sex and that there may be a cohort effect among girls. This likely represents differences in diagnostic patterns which should be investigated to see if this phenomenon has implications on management and morbidity outcomes. We also conclude that adolescents exposed to passive smoking exposure are at increased risk of developing asthma and smoking avoidance should be practiced. Finally, we did not find hypothesized associations between asthma and rural dwelling or health behaviours. This likely indicates a need for further research into potential interactions or into the underlying causal exposures such as farming, or timing of exposures.

## Abbreviations

BMI: Body Mass Index; CI: Confidence Interval; HR: Hazard Ratio; Kcal: Kilo-calorie; Kg: Kilogram; m: Meter; NPHS: National Population Health Survey; P: Probability value; RDC: Research Data Center; US: United States; Vs: Versus.

## Competing interests

The authors declare that the have no competing interests.

## Authors’ contributions

JL coordinated this research including developing the hypotheses and research direction, analysis planning, coordinating access to data and initial drafting of the manuscript; IJ was involved in the original planning of the research plan specific to this analysis and the interpretation and editing of the manuscript; MB was involved in the original planning of the research plan specific to this analysis and the interpretation and editing of the manuscript; AH was the analyst who helped develop the analysis plan, completed the data analysis, and aided in the interpretation and editing of the manuscript. WP was involved in the original planning of the research plan specific to this analysis and the interpretation and editing of the manuscript. All authors read and approved the final manuscript. We would also like to acknowledge the participants of this study for their commitment to this important longitudinal study and investment of time to this project.

## Pre-publication history

The pre-publication history for this paper can be accessed here:

http://www.biomedcentral.com/1471-2466/14/51/prepub
